# Complementary and Alternative Medicine Use Among Patients With Diabetes Mellitus in Saudi Arabia: A Community-Based Cross-Sectional Study

**DOI:** 10.7759/cureus.45792

**Published:** 2023-09-22

**Authors:** Khaled A Yaghmour, Raneem Abu Sadi, Ftoon Badroun, Rezan Alali, Fatimah Almubarak, Zainab Alabbad, Noura Alharthi, Jamil A Samkari, Mahmoud A Gaddoury

**Affiliations:** 1 Family Medicine Department, King Abdulaziz University, Jeddah, SAU; 2 Medical Intern, King Abdulaziz University Faculty of Medicine, Jeddah, SAU; 3 Medical Intern, King Abdulaziz University, Jeddah, SAU; 4 Family and Community Medicine, King Abdulaziz University Faculty of Medicine, Rabigh, Jeddah, SAU; 5 Community Medicine, King Abdulaziz University, Faculty of Medicine, Jeddah, SAU

**Keywords:** complementary and alternative, cross-sectional, community-based, saudi, patients, diabetes mellitus

## Abstract

Background: Global studies have observed a disparity in the use of complementary and alternative medicine (CAM) among diabetic patients.

Objectives: To estimate the prevalence, types, and correlates of CAM use among patients with diabetes mellitus (DM) in Saudi Arabia.

Methodology: A cross-sectional study was conducted among 1290 Saudis with type 2 DM aged ≥18 years. An electronic questionnaire was distributed through social media to collect data about patient demographics and DM-related characteristics, including age at DM diagnosis, DM duration, family history of DM, DM complications, DM medicine, and chronic diseases. The use of CAM and its type, cost, and duration; sources of CAM-related information; reason for using CAM; usefulness and side effects; CAM use in the future; and doctor consultation before CAM use were also evaluated. Among CAM non-users, the reason for not using CAM and future considerations of CAM were assessed.

Results: A total of 1290 patients were included (27.4%) aged 18-29 years; 726 (56.3%) were women; 554 (42.9%) had a bachelor’s degree in education; and 457 (35.4%) were unemployed. The prevalence of CAM use was 528 (40.9%). The most commonly used types were bitter apple 503 (95.3%), cinnamon 341 (64.6%), and ginger 290 (55.1%). The most frequent sources of CAM-related information were friends, families, and neighbors 259 (49.2%), while the most frequent justifications for use were the need for another DM treatment and faith in its advantages. Only 106 (20.1%) of the patients who used CAM disclosed adverse effects; 373 (51.8%) said they would use it again, and 66.1% said they would recommend it to other patients. Only 145 (27.5%) consulted a doctor before using CAM. CAM was more commonly used by patients who were older, women, married, and taking hypoglycemic drugs; whose most recent HbA1c level was 7-10%; and who had dyslipidemia, chronic disease, and a family history of DM.

Conclusion: The prevalence of CAM use is high among the Saudi population. Analyzing CAM use is essential in clinical interactions with Saudis with DM. The managing healthcare professionals must educate patients with DM on how to use CAM more effectively and safely.

## Introduction

Diabetes mellitus (DM) is described as a “chronic disease that develops when the body is unable to use the insulin that is produced or when the pancreas does not produce enough insulin.” An insulin-independent variant of DM (type 2 DM) develops when there is a state of insulin resistance in our body [1]. Uncontrolled hyperglycemia over the years leads to microvascular complications like diabetic nephropathy, neuropathy, and retinopathy and could further lead to macrovascular complications like heart disease, stroke, etc. [1,2]. Diabetes mellitus is a risk factor for cardiovascular disease (CVD) and has been associated with twofold to fourfold higher mortality [2].

Approximately 537 million adults (20-79 years old) will be living with diabetes in 2022, and by 2045, this number is projected to increase to 693 million [3]. Saudi Arabia has the second-highest rate of DM after Kuwait in the Middle East and the seventh-highest rate worldwide, with approximately 34% of the population having the disease [4,5].

Traditional and complementary medicine is frequently used worldwide, especially by patients with chronic illnesses such as DM [6-9]. The World Health Organization describes complementary and alternative medicine (CAM) as “a broad set of health care practices that are not part of that country’s own tradition or conventional medicine and are not fully integrated into the dominant health care system" [7].

In 2021, researchers conducted a global systematic review and meta-analysis of 38 studies on the frequency and types of CAM use among patients with DM. The studies evaluated the prevalence and utilization of CAM among patients with DM who were at least 18 years old. The results showed a prevalence of CAM use among patients with DM ranging from 8% to 89%, with a combined prevalence of 51%. Along with 223 different kinds of herbs, 37 different varieties of CAM were found to be used. The most frequently reported CAM types were acupuncture, homeopathy, spiritual healing, and herbal remedies. A third of patients did not disclose their use of CAM to healthcare professionals [8].

In Saudi Arabia, a descriptive review was conducted among 36 articles on national surveys carried out from 2000 to 2015. The results showed a substantial range in CAM use patterns of 21.6-90.5%. Although women made up the majority of users, the study discovered a significant difference in CAM practice between male and female users. The most popular spiritual activities were praying and reciting the Quran aloud or while floating. The other types of CAM included herbs (8-76%), honey (14-73%), and dietary supplements (6-82%). The least frequently used method was cupping (4-45%). Medical professionals use acupuncture more frequently [2].

The use of CAM among patients with DM was examined in a further Saudi descriptive review in 2018, with a focus on determining the prevalence and discussing the safety and efficacy of this treatment. The results showed that the prevalence of the use of CAM ranged from 17.4% to 64%, with herbs and honey being the most popular CAM treatments. The prevalence is similar among Saudi Arabia and India (67.8%) [9]. Saudi Arabia ranks fourth among Arab nations that use CAM for patients with DM. Additionally, in the 2018 study, the majority of traditional and complementary medicine users failed to disclose their use of CAM to their doctors because physicians did not ask them about their use of T&CM modalities [10].

In Taif, a cross-sectional study was conducted to determine the prevalence and predictors of CAM use in patients with DM. The study found that 33.7% of people used CAM, with 87.3% not consulting a doctor in the first place. Approximately 43.2% of them used more than one source of information, and 62.7% used more than one type of CAM. Around 49.2% and 72.9% found CAM to be extremely useful and to have no negative side effects, respectively. Furthermore, 47.5% of patients with DM would recommend CAM to other patients. A cent percent of participants used the bitter apple as their complementary medicine, followed by cinnamon (66.1%), ginger (55%), ginger (35.6%), fenugreek (21.2%), and garlic. The increased use of CAM was associated with female gender, family history of DM, DM complications, and longer duration of DM [3].

There are insufficient community-based studies to determine the prevalence of CAM use among Saudi patients with DM. Accordingly, the findings of the present study will provide information that decision-makers can use to determine whether CAM should be used in conjunction with conventional therapy. This can maximize the benefit to patients while avoiding medical problems due to side effects and drug interactions.

The present study aimed to estimate the prevalence and assess the type and correlates of CAM use among patients with DM in a Saudi Arabian community.

## Materials and methods

Study design, setting, and time

This cross-sectional survey was conducted in all regions of Saudi Arabia for three months, from February to April 2023.

Study participants

Saudis diagnosed with type 2 DM aged ≥18 years were included, while patients aged under 18 years, other types of diabetes, and pregnant women were excluded.

Sample size

The sample size was calculated using the Raosoft sample size calculator (http://www.raosoft.com/samplesize.html). The required sample size was estimated at a confidence level of 95%, a total population size of 35,361,026 [11], a margin of error of ±5%, and an assumed prevalence of 50%. The required minimum sample size was determined to be 385.

Data collection

An electronic questionnaire was distributed through social media to collect data. (i) The first section of the questionnaire collected data about the participant demographics (age, gender, nationality, region, marital status, monthly income, educational level, and occupation). (ii) The second section collected data about DM-related characteristics, including age at DM diagnosis, duration of DM, family history of DM, presence of DM complications, DM medicine used regularly, and other chronic diseases. (iii) The third section assessed the participants’ use of CAM. Among CAM users, the CAM use pattern was assessed (type, cost, duration of use, sources of information, reason for using CAM, expectation when using CAM, feeling after using CAM, usefulness of CAM, side effects of CAM, re-use of CAM, recommendation of CAM to other patients with DM, and consultation with a doctor before using CAM). Among CAM non-users, the reason for not using CAM and consideration of CAM in the future were assessed.

Ethical considerations

Ethical approval for the study was obtained from the research ethics committee of the Faculty of Medicine, King Abdulaziz University, Jeddah, Saudi Arabia (Reference No. 339-22). Participants were informed that the study will not collect any identifying information, and all responses will be kept confidential.

Data analysis

Data were analyzed using the SPSS program version 26 (Armonk, NY: IBM Corp.). Qualitative data were expressed as numbers and percentages, and the χ2 test was used to assess the relationship between variables. Quantitative data were expressed as means and SDs and checked for normality. Non-parametric variables were tested using the Mann-Whitney test and presented as medians (IQR). A p-value of <0.05 was considered statistically significant.

## Results

A total of 1290 participants were included. Table 1 shows that 27.4% of the participants were aged 18-29 years; 56.3% were women; 93.3% had a Saudi nationality; and 35% were from the southern region. Of them, 42.9% had a bachelor’s degree in education, and 35.4% were unemployed.

**Table 1:** Distribution of the participants according to their demographic characters (N=1290)

Variable	No. (%)
Age (years)	
18–29	354 (27.4)
30-39	196 (15.2)
40–49	241 (18.7)
50–59	315 (24.4)
60–70	132 (10.2)
>70	52 (4)
Gender	
Female	726 (56.3)
Male	564 (43.7)
Nationality	
Saudi	1204 (93.3)
Non-Saudi	86 (6.7)
KSA region	
Eastern province	84 (6.5)
Centra	250 (19.4)
Northern	155 (12)
Southern	451 (35)
Western	350 (27.1)
Marital status	
Not married	403 (31.2)
Married	887 (68.8)
Monthly income (Saudi Rials)	
<5000	344 (26.7)
5000-<10,000	370 (28.7)
10,000-15,555	327 (25.3)
>15,000	249 (19.3)
Educational level	
Illiterate	94 (7.3)
Primary	56 (4.3)
Middle	62 (4.8)
Secondar	266 (20.6)
Bachelor	554 (42.9)
Master	72 (5.6)
PhD	24 (1.9)
Occupation	
Employed	330 (25.6)
Retired	221 (17.1)
Unemployed	457 (35.4)
Students/homemaker	282 (21.9)

Table 2 demonstrates that 45.7% of the participants were on oral hypoglycemic drugs, and 52.3% reported that they were committed to their DM treatment. Most of the participants (36.3%) reported that their cumulative blood sugar level in the last three months ranged from 7% to 10%. Around 29.5% of the participants had a chronic disease other than DM, and the most common comorbidity was hypertension (HTN; 80.5%). Approximately 69.8% had an FH of DM, and 20.5% had DM complications, with diabetic retinopathy (68.6%) being the most common.

**Table 2:** Distribution of the participants according to diabetes mellitus clinical data (no.:1290)

Variable	No. (%)
What was your age when you were diagnosed with diabetes?	31.34 ± 14.99
What treatment are you currently using for diabetes?	
Hypoglycemic drug	589 (45.7)
Hypoglycemic drugs + insulin	280 (21.7)
Insulin	421 (32.6)
How committed are you to treatment? (adherence to a prescribed diet and treatment)	
Uncommitted	64 (5)
Somewhat committed	440 (34.1)
Very committed	675 (52.3)
Little committed	111 (8.6)
In the last 3 months, what is the result of the last HbA1c analysis?	
<7%	375 (29.1)
7–10%	468 (36.3)
>10%	83 (6.4)
NA	45 (3.5)
Do you have other chronic diseases (such as pressure, heart disease, etc.? If you answered yes, what other chronic diseases do you have?	
No	910 (70.5)
Yes	380 (29.5)
If having other chronic diseases, specify (no.: 380):	
Cardiac disorders	34 (8.9)
HTN	306 (80.5)
Dyslipidemia	26 (6.8)
Thyroid disorders	10 (2.6)
Asthma	4 (1)
Stroke	2 (0.5)
Musculoskeletal disorders	9 (2.3)
Do you have anyone in the family history of diabetes?	
No	389 (30.2)
Yes	901 (69.8)
Do you have any complications from diabetes?	
No	1025 (79.5)
Yes	265 (20.5)
If having complications, specify (no.:265):	
Retinopathy	182 (68.6)
Nephropathy	83 (3)
Coronary heart disease	102 (38.4)
Cerebrovascular disease	10 (3.7)

**Figure 1:**
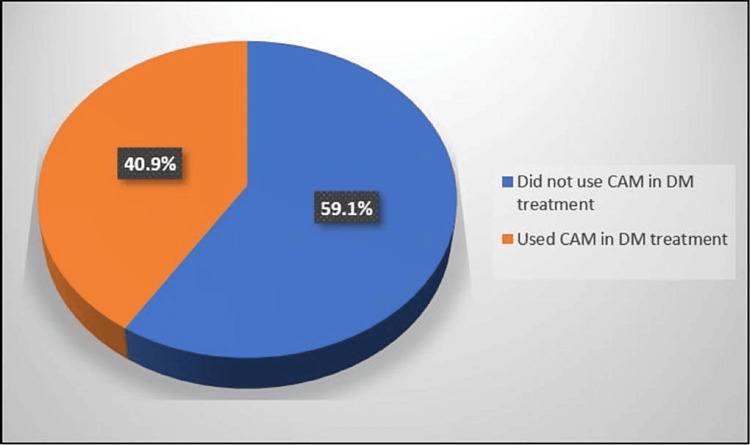
Percentage distribution of the participants according to complementary and alternative medicine use to treat diabetes since being diagnosed with diabetes (no.:1290)

**Figure 2:**
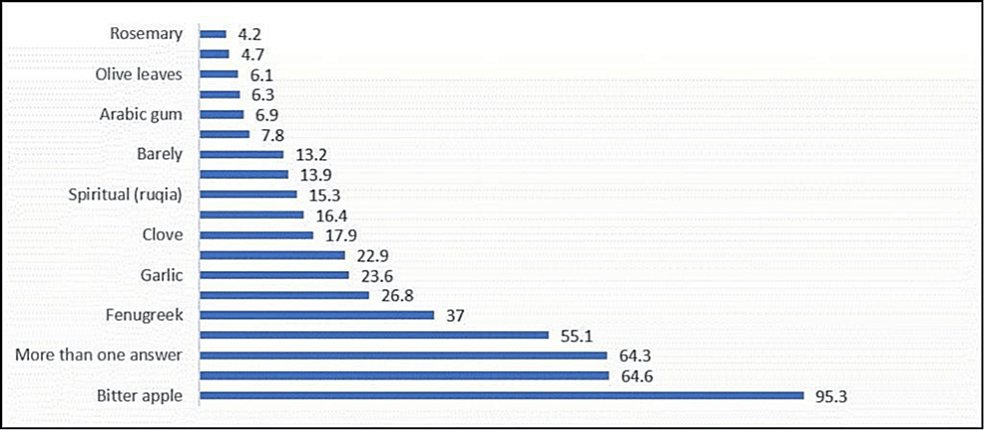
Percentage distribution of complementary and alternative medicine type among complementary and alternative medicine users (no.:528) N.B.: More than one answer was allowed

Of the patients with DM, 528 (40.9%) have used CAM for DM treatment since diagnosis (Figure 1). Among the CAM users, the most common CAM types used were bitter apple 503 (95.3%), cinnamon 341 (64.6%), and ginger 290 (55.1%) (Figure 2).

The median (IQR) cost of CAM was 120,350 SR, and 21.2% of the patients had been using CAM for months. Approximately 66.5% used CAM for the treatment of DM in the last year. The most common sources of CAM-related information were friends, relatives, and neighbors (49.2%). The most common expectations when using CAM were a reduction in the level of sugar in the blood (61.1%) and better health (51.1%). The most commonly reported reasons for using CAM were the search for a solution or other DM treatment (62.2%) and belief in the benefits of CAM (52.8%). Approximately 38.8% of the users felt in a good psychological state after using CAM, but 39.3% rated CAM as being of limited benefit. Only 20.1% noticed side or adverse effects of CAM, and 51.8% were willing to use CAM in the future. The majority of the users (66.1%) would recommend CAM to other patients with DM, and only 27.5% consulted a doctor before using CAM (Table 3).

**Table 3:** Distribution of complementary and alternative medicine users according to pattern and experience of complementary and alternative medicine use (No.:528)

Variable	No. (%)
For CAM users (No.:528)	
The cost of used CAM (SR)	351.83 ± 681.3
The duration for which the CAM is used	
Weeks	75 (14.2)
Months	112 (21.2)
Days	100 (18.9)
Years	37 (7)
NA	241 (45.6)
Have you used the CAM in the treatment of diabetes last year?	
No	177 (33.5)
Yes	351 (66.5)
Sources from which I got information about CAM	
Personal choice	144 (27.2)
Family beliefs	174 (32.9)
Health care providers	26 (4.9)
Friends, relatives, and neighbors	260 (49.2)
Media and social networking	126 (23.8)
Internet	179 (33.9)
What are your expectations of the CAM when using it?	
Better health	270 (51.1)
Weight loss	81 (15.3)
No expectations	64 (12.1)
Preventing the aggravation of diabetes	182 (34.4)
Reducing the level of sugar in the blood	323 (61.1)
Why use CAM?	
Belief and belief in the benefits of CAM	279 (52.8
Search for a solution or other DM treatment	318 (60.2)
CAMs are available and can be obtained	223 (42.2)
I lost hope in modern treatments	73 (13.8)
How do you feel after using the CAM?	
I feel that I am in a bad physical condition	14 (2.6)
I feel that I am in a bad psychological condition	16 (3)
I am afraid of the product and its side or adverse effects	28 (5.3)
I feel the emergence of new symptoms of the disease	17 (3.2)
Feeling that you are in a good psychological state	205 (38.8)
No change	99 (18.7)
How would you rate the usefulness of a CAM?	
Not sure unable to rate	161 (30.4)
Not useful at all	29 (5.4)
Of limited benefit	208 (39.3)
Very useful	130 (24.6)
Have you noticed any side effects or adverse effects of the CAM after using it?	
No	422 (79.9)
Yes	106 (20.1)
Will you use CAM in the future?	
No	255 (48.2)
Yes	373 (51.8)
Would you recommend this CAM for other diabetics?	
No	179 (33.9)
Yes	349 (66.1)
Have you consulted a doctor before using a CAM?	
No	383 (72.5)
Yes	145 (27.5)

Among the non-users, the most common reasons for not using CAM were non-prescription by a doctor (32.9%) and non-belief in the benefits of CAM (23.6%) (Table 4). Only 21% of the non-users were willing to use CAM in the future.

**Table 4:** Distribution of complementary and alternative medicine non-users according to reasons for not using complementary and alternative medicine and willing to use it in the future (no.:726)

Variable	No. (%)
For CAM non-users (No.:762)	
What are the reasons for not using CAM?	
CAM is not based on scientific evidence	81 (10.6)
No one recommended its use	152 (19.9)
Afraid of side and adverse effects	140 (18.3)
Because modern medicine is the best	142 (18.6)
Because the doctor did not prescribe it to me	251 (32.9)
Not interested in CAM	117 (15.3)
I don't believe in CAM	180 (23.6)
Are you thinking of using CAM in the future?	
No	602 (79)
Yes	160 (21)

Table 5 shows that the prevalence of CAM use was significantly higher among the participants who were aged from 50 to 59 years, women, from the southern KSA region, and married (p≤0.05).

**Table 5:** Relationship between complementary and alternative medicine use and participants' demographics characters (no.:1290)

Variable	CAM use		χ2	p-value
	No No. (%)	Yes No. (%)		
Age (years)				
18–29	236 (31)	118 (22.3)	16.85	0.005
30–39	108 (14.2)	88 (16.7)		
40-49	147 (19.3)	94 (17.8)		
50-59	169 (22.2)	146 (27.7)		
60-70	69 (9.1)	63 (11.9)		
>70	33 (4.3)	19 (3.6)		
Gender				
Female	409 (53.7)	317 (60)	5.13	0.023
Male	353 (46.3)	211 (40)		
Nationality				
Saudi	798 (92.9)	496 (93.9)	0.52	0.568
Non-Saudi	54 (7.1)	32 (6.1)		
KSA region				
Eastern province	55 (7.2)	29 (5.5)	22.39	<0.001
Central	136 (17.8)	114 (21.6)		
Northern	114 (15)	41 (7.8)		
Southern	271 (35.6)	180 (34.1)		
Western	186 (24.4)	165 (31.1)		
Marital status				
Not married	258 (33.9)	145 (27.5)	5.94	0.015
Married	504 (66.1)	383 (72.5)		
Monthly income (SR)				
<5000	212 (27.8)	132 (25)	2.28	0.516
5000–<10,000	222 (29.1)	148 (28)		
10,000–15,555	184 (24.1)	143 (27.1)		
>15,000	144 (18.9)	105 (19.9)		
Illiterate	61 (8)	33 (6.3)	7.12	0.416
Primary	31 (4.1)	25 (4.7)		
Middle	30 (3.9)	32 (6.1)		
Secondar	160 (21)	106 (20.1)		
Bachelor	327 (42.9)	227 (43)		
Master	38 (5)	34 (6.4)		
PhD	13 (1.7)	11 (2.1)		
Occupation				
Employed	190 (24.9)	140 (26.5)	1.65	0.064
Retired	114 (15)	107 (20.3)		
Unemployed	278 (36.5)	179 (33.9)		
Students/homemaker	180 (23.6)	102 (19.3)		

CAM use was also significantly more common among the participants who were on hypoglycemic drugs for DM management, who reported that they were committed to DM treatment, whose cumulative blood sugar level in the last three months ranged from 7% to 10%, and who had dyslipidemia as a comorbidity (p≤0.05) (Table 6).

**Table 6:** Relationship between complementary and alternative medicine use and participants' diabetes mellitus clinical data (no.:1290)

Variable	CAM use		χ2	p-value
	No No. (%)	Yes No. (%)		
What was your age when you were diagnosed with diabetes?	30.69 ± 14.99	32.28 ± 14.96	1.77	0.077
What treatment are you currently using for diabetes?				
Hypoglycemic drugs	339 (44.5)	250 (47.3)	16.35	<0.001
Hypoglycemic drugs+ insulin	144 (18.9)	136 (25.8)		
Insulin	279 (36.6)	142 (36.9)		
How committed are you to treatment?				
Uncommitted	49 (6.4)	15 (2.8)	13.21	0.004
Somewhat committed	238 (31.2)	202 (38.3)		
Very committed	408 (53.5)	267 (50.6)		
Little committed	67 (8.8)	44 (8.3)		
In the last 3 months, what is the result of the last cumulative blood sugar analysis?				
<7%	224 (29.4)	151 (28.6)	11.66	0.02
7%-10%	262 (34.4)	206 (39)		
>10%	56 (7.3)	27 (5.1)		
NA	19 (2.5)	26 (4.9)		
If having other chronic disease, specify (No.:380):				
Cardiac disorders	18 (2.4)	16 (3)	0.54	0.461
HTN	168 (22)	138 (26.1)	2.88	0.09
Dyslipidemia	10 (1.3)	16 (3)	4.66	0.03
Thyroid disorders	7 (0.9)	3 (0.6)	0.49	0.48
Asthma	2 (0.3)	2 (0.4)	0.13	0.712
Stroke	1 (0.1)	1 (0.2)	0.06	0.794
Musculoskeletal disorders	4 (0.5)	5 (0.9)	0.8	0.371
Do you have any complications from diabetes?				
No	146 (19.2)	119 (22.5)	2.18	0.14
Yes	616 (80.8)	409 (77.5)		
If having DM complications, specify (No.:1025):				
Retinopathy	102 (13.4)	80 (15.2)	0.8	0.37
Nephropathy	53 (7)	30 (5.7)	0.84	0.35
Coronary heart disease	55 (7.2)	47 (8.9)	1.21	0.271
Cerebrovascular disease	5 (0.7)	5 (0.9)	0.34	0.558

**Figure 3:**
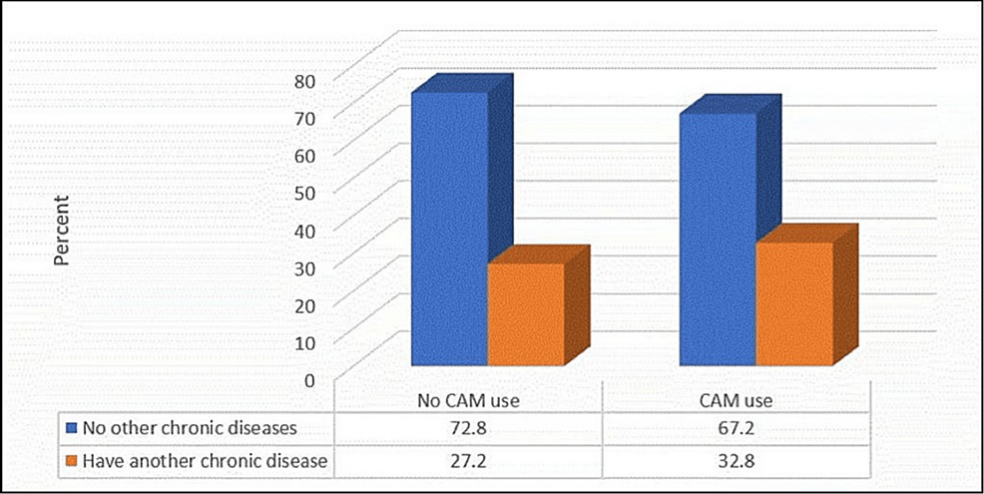
Relationship between complementary and alternative medicine use and having a chronic disease other than diabetes mellitus (no.:1290) N.B.: (χ2 = 4.7, p-value = 0.03)

**Figure 4:**
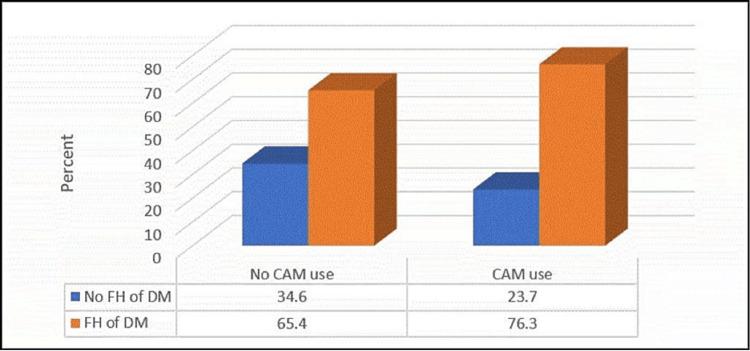
Relationship between complementary and alternative medicine use and having a family history of diabetes (no.:1290) N.B.: (χ2 = 17.82, p-value ≤ 0.001)

Figures 3-4 demonstrate that CAM use was significantly more common among the participants with other chronic diseases and an FH of DM (p≤0.05).

## Discussion

The purpose of this study was to estimate the prevalence and identify the types and correlates of CAM use among patients with DM in Saudi Arabia. Of the participants, 40.9% used CAM. This prevalence is consistent with that reported in a previous systematic review of 18 studies from 9 countries conducted among patients with DM, which found a prevalence that ranged from 17.2% to 72.8% [10,12]. The present finding also supports the data from a Saudi Arabian systematic review of 36 articles published from 2000 to 2015, where CAM use was prevalent among 21.6-90.5% of the general population [2]. Another Saudi study conducted in 2018 found that the prevalence of CAM use among patients with DM had a wide range of 17.4% to 64% [9].

According to earlier national studies, the prevalence of CAM use among patients with DM in Riyadh, Saudi Arabia, was 30.5% in 2016 and 32.18% in 2018 [10,13]. In Taif, the prevalence was 24.6% in a 2014 study [14], whereas 33.7% of patients with DM used CAM in a 2020 study [3]. A prevalence of 30.1% was noted in the Mecca region of Saudi Arabia [15].

In comparison with a Malaysian study (62.5%), CAM use among patients with DM was lower in Saudi Arabia [16]. And when comparing the Saudi prevalence with Gulf countries, a higher prevalence was reported in Bahrain [17]. This variation could be attributed to the various study designs, sociocultural perceptions of CAM use, availability, accessibility, and types of CAM. Such differences could have affected the prevalence reported in various studies.

In this study, 95.3% of CAM users used bitter apples, whereas more than 50% of them used cinnamon and ginger. Similar findings were found in an earlier Saudi study, wherein 100% of patients with DM were using bitter apple; 66.1%, cinnamon; 55.1%, ginger; 35.6%, fenugreek; and 21.2%, garlic, as their only CAM. A nearly identical proportion of patients (62.7%) were using multiple CAM techniques [3]. Alrowais and Alyousefi [2] discovered that patients used primarily myrrh (Commiphora molmol), black seeds (Nigella sativa), fenugreek (Trigonella foenum-graecum), helteet (Ferula assa-foetida), and aloe (Aloe vera) in their earlier survey.

Friends, families, and neighbors were the most frequent sources of information about CAM among the participants in the present study. In previous research, it was discovered that friends and peers, rather than doctors and chemists, were frequently consulted when using CAM [14]. Additionally, a previous Taif study discovered that 43.2% of patients with DM had access to more than one source of CAM-related information [3].

According to the patients in the current study, seeking a cure or another DM treatment (62.2%) and having faith in CAM’s potential benefits (52.8%) were the main motivators for using CAM. Another study discovered that the majority of patients with DM who developed retinopathy or other complications used CAM to lessen their symptoms and prevent side effects [18]. Previous research has also found that easy accessibility, greater affordability, strong belief in the efficacy, and fewer perceived side effects associated with CAM in comparison with prescription drugs were the main drivers behind its use among patients with DM [19,20].

The current findings on the usefulness of CAM use showed that while 39.3% of the participants felt CAM to be of limited value, 38.8% reported feeling in a good psychological state after using it. This level of satisfaction with CAM use is in line with the results of numerous international studies on CAM-related behaviors and attitudes [21].

In the current study, only 20.1% of the patients with DM reported experiencing negative side effects from CAM, and nearly half (51.8%) said they would be willing to use it again. In a previous Saudi study, 49.2% of participants said CAM was extremely helpful, and 72.9% said there were no negative effects from using it [3].

Most CAM users (66.1%) in the present study said they would suggest it to other patients with DM. Both national and international studies have drawn the same conclusion [3,16].

In the current study, only 27.5% of the participants sought medical advice before using CAM. In a previous Saudi study [3], only 12.7% of patients with DM consulted a physician before using CAM. This outcome is consistent with earlier Saudi reports, wherein only a minority sought medical advice prior to using CAM [2,16]. This finding demonstrates how a poor doctor-patient (and pharmacist-patient) relationship causes patients to hide their CAM use. This emphasizes the role of the treating physician to ask in detail about any CAM used by the patient.

Herein, the use of CAM was significantly more frequent among the patients with DM who were older, were married, were taking hypoglycemic medication, had an FH of DM, had a blood sugar level in the last 3 months of 7-10%, and had other chronic diseases, particularly dyslipidemia. These findings are consistent with another Saudi study, where female gender, FH of DM, DM complications, and longer duration of DM were linked to increased use of CAM in previous Saudi studies [3,22,23].

It has been discovered that patients may feel pressured to seek CAM treatment if a family member uses it or if they believe a complication left untreated by conventional medicine exists. The affordability of CAM has also been proven to encourage its use [24].

## Conclusions

This study found a high prevalence of CAM use among studied DM patients. Less than one-third sought medical advice before using CAM. The use of CAM was significantly higher among older patients, women, married, those on hypoglycemic medications, those with uncontrolled DM, as well as among patients with chronic disease, dyslipidemia, and FH of DM. Decision-makers should weigh the risks and benefits of complementary and alternative therapies. A concerted effort by the government, orders and syndicates, medical, nursing, and health schools, and educational institutions are required to improve education about the safe use of CAM and to encourage diabetic patients to share their use with their family physicians. There is a need to bridge traditional and modern healthcare practices in the context of diabetes management. In addition, cohort studies are recommended to assess the described causal relationships between variables.
